# A Novel Ferroelectric Rashba Semiconductor

**DOI:** 10.1002/adma.202310278

**Published:** 2023-12-24

**Authors:** Gauthier Krizman, Tetiana Zakusylo, Lakshmi Sajeev, Mahdi Hajlaoui, Takuya Takashiro, Marcin Rosmus, Natalia Olszowska, Jacek J. Kołodziej, Günther Bauer, Ondrej Caha, Gunther Springholz

**Affiliations:** ^1^ Institut für Halbleiter und Festkörperphysik Johannes Kepler Universität Altenberger Strasse 69 Linz 4040 Austria; ^2^ Department of Condensed Matter Physics Masaryk University Kotlárská 2 Brno 61137 Czech Republic; ^3^ National Synchrotron Radiation Centre SOLARIS Jagiellonian University Czerwone Maki 98 Krakow 30‐392 Poland; ^4^ Faculty of Physics Astronomy and Applied Computer Science Jagiellonian University Ul. Prof. Stanislawa Lojasiewizca 11 Krakow 30–348 Poland

**Keywords:** angle‐resolved photoemission spectroscopy, ferroelectricity, IV‐VI compounds, phase transition, rashba spin texture, x‐ray diffraction

## Abstract

Fast, reversible, and low‐power manipulation of the spin texture is crucial for next generation spintronic devices like non‐volatile bipolar memories, switchable spin current injectors or spin field effect transistors. Ferroelectric Rashba semiconductors (FERSC) are the ideal class of materials for the realization of such devices. Their ferroelectric character enables an electronic control of the Rashba‐type spin texture by means of the reversible and switchable polarization. Yet, only very few materials are established to belong to this class of multifunctional materials. Here, Pb_1−_ *_x_* Ge*_x_* Te is unraveled as a novel FERSC system down to nanoscale. The ferroelectric phase transition and concomitant lattice distortion are demonstrated by temperature dependent X‐ray diffraction, and their effect on electronic properties are measured by angle‐resolved photoemission spectroscopy. In few nanometer‐thick epitaxial heterostructures, a large Rashba spin‐splitting is exhibiting a wide tuning range as a function of temperature and Ge content. This work defines Pb_1−_ *_x_* Ge*_x_* Te as a high‐potential FERSC system for spintronic applications.

## Graphical abstract

A ferroelectric Rashba semiconductor owns a definite Rashba spin‐texture that can be manipulated and reversed by an applied electric field. Such a material is highly sought‐after for spintronic transistors and/or memories. This work presents an original demonstration of a new ferroelectric Rashba semiconductor, by confronting its measured structural ferroelectric distortion to its observed Rashba electronic properties.

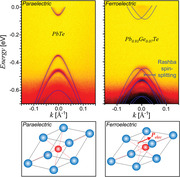

## 1 Introduction

**Figure 1:**
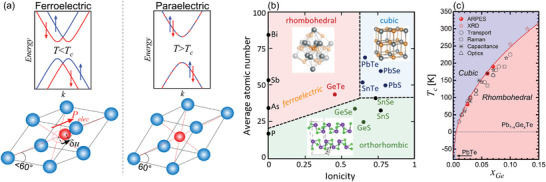
IV‐VI and Pb_1_ *_−x_* Ge*_x_* Te systems as FERSC. a) Comparison of the electronic band structure of a rhombohedral distorted FE semiconductor such as GeTe (left) with that of a paraelectric (PE) cubic semiconductor such as PbTe (right). As shown in the lower panel, in the rhombohedral phase, the cations (red) are shifted by δ*u* with respect to the anions (blue) along the <111> direction. This induces an electric dipole responsible for a Rashba‐type spin splitting of the bands illustrated in the top panels. In the cubic phase, shown on the right, the centrosymmetric cation site is located at the center of the anionic sublattice and thus, the electric dipole is zero and the bands are Kramer's spin degenerate. b) Polymorphism in IV‐VI class of compounds illustrating the formation of different structural phases and ferroelectricity as a function of ionicity and average number of electrons per atom (after Ref. [57]). c) Curie temperature of Pb_1_ *_−x_* Ge*_x_* Te, showing the FE phase transition as a function of temperature. In red are the data points resulting from this work, which are compared with literature values (in black) obtained by transport,^[^ ^51^ ^]^ capacitance,^[^ ^50^, ^58^ ^]^ optical,^[^ ^53^, ^56^, ^59^ ^]^ X‐ray,^[^ ^60^ ^]^ and Raman^[^ ^61^ ^]^ measurements. The solid line is a fit to our experimental data, giving $T_{c}\;\left(x\right)=\;1000\sqrt{x}-70$ [K].

Ferroelectric Rashba semiconductors (FERSC) have been recently disclosed as a new class of multifunctional materials to enrich electronic and spintronic device technologies.^[^ ^1^, ^2^, ^3^, ^4^, ^5^, ^6^, ^7^, ^8^, ^9^ ^]^ The unique feature of FERSC is the fundamental breaking of the inversion symmetry caused by a ferroelectric (FE) lattice distortion, which leads to a large spin splitting of the electronic band structure in k‐space by the Rashba effect^[^ ^10^, ^11^ ^]^ (**Figure** **1**a). The direction of the spin polarization, that is, the helicity of the spin texture is locked to the FE polarization. This means that in a FERSC, the spin polarization can be externally controlled and reversed by an applied electric field via a non‐volatile and switchable poling process.^[^ ^12^, ^13^ ^]^ This remarkable property is singular to this class of multifunctional materials and is sought‐after for spintronic applications such as spin field effect transistors, non‐volatile and bipolar memories as well as programmable transistors for nematics and logic operations.^[^ ^8^, ^13^, ^14^, ^15^, ^16^ ^]^

The development of FERSC demands materials exhibiting ferroelectricity, semiconductor properties and a sizeable Rashba effect at the same time. Recent theoretical studies have suggested a number of potential FERSC candidates like complex oxides,^[^ ^17^, ^18^, ^19^ ^]^ perovskites^[^ ^20^, ^21^, ^22^ ^]^ or 2D materials,^[^ ^15^, ^23^, ^24^ ^]^ but so far, FERSC have been demonstrated experimentally only for the IV‐VI class of semiconductors (see Figure 1b). The key representative is α‐GeTe,^[^ ^3^, ^4^, ^12^, ^13^, ^25^, ^26^, ^27^, ^28^, ^29^ ^]^ which is FE below its Curie temperature of *T_c_* ≈ 700 K^[^ ^30^ ^]^ and displays a giant Rashba effect.^[^ ^12^, ^25^, ^27^, ^28^, ^29^, ^31^, ^32^ ^]^ This is due to the large rhombohedral lattice distortion in which the cation Ge^2+^ and anion Te^2−^ sublattices^[^ ^30^ ^]^ are shifted with respect to each other by as much as 0.3 Å along the <111> direction,^[^ ^33^ ^]^ as illustrated by Figure 1a. This induces a permanent electric dipole that accounts for a macroscopic FE polarization and the concomitant Rashba effect on the electronic band structure. Moreover, by means of an applied electric field, a switching of the spin polarization by controlling the FE polarization has been demonstrated.^[^ ^34^ ^]^ Although a giant Rashba effect has been also observed for the highly polar compounds BiTeI^[^ ^35^, ^36^ ^]^ and BiTeBr,^[^ ^37^ ^]^ because these are non‐ferroelectric, the spin polarization in them cannot be switched and reversed permanently by an electric field.

The drawback of α‐GeTe for FERSC applications is its intrinsic high p‐type conductivity that arises from the high density of electrically active Ge vacancies in the crystal lattice. This results in a rather large intrinsic hole concentration above 10^20^ cm^−3^ that cannot be compensated,^[^ ^4^, ^38^ ^]^ which impedes efficient electrical control of the FE polarization due to leakage currents. In addition, α‐GeTe has an indirect band gap,^[^ ^39^ ^]^ which makes it not suitable for optical devices. Last but not least, the bulk FE Rashba effect of α‐GeTe is superimposed and partially screened by the giant Rashba effect of the localized surface states^[^ ^27^, ^40^ ^]^ caused by the tellurium surface termination favored by the free surface energy of the system. Thus, for α‐GeTe the experimental identification of the bulk FE Rashba effect requires detailed analysis of photoemission spectroscopy data by ab initio density functional theory calculations to sort out between the intrinsic bulk and the extrinsic surface Rashba effects. It is noted that SnTe (Figure 1b) is another potential candidate for FERSC, but it suffers from the same problems as α‐GeTe (except for the indirect gap). Moreover, SnTe exhibits a relatively low *T_c_* ≈ 100 K^[^ ^41^ ^]^ and thus, does not provide a solution for device applications.^[^ ^13^, ^31^, ^42^ ^]^

In this work, we pursue an alternative approach to overcome the limitations of α‐GeTe, based on the conversion of PE PbTe by GeTe doping to a FERSC with superior properties for device applications. PbTe is a versatile IV‐VI compound that has a direct band gap, as well as several orders of magnitude lower carrier concentration as compared to GeTe and SnTe. Moreover, it features very high carrier mobilities exceeding 10^6^ cm^2^ V^−1^ s^−1^ at low temperatures^[^ ^43^ ^]^ and can be effectively doped *p*‐ as well as *n*‐type.^[^ ^44^ ^]^ Due to the more ionic character compared to GeTe, PbTe crystallizes in the centrosymmetric cubic rock salt structure. However, it is very close to a PE‐FE phase transition (see Figure 1b), which is signified by the pronounced transverse optical phonon softening^[^ ^45^, ^46^ ^]^ and strong increase of the dielectric constant^[^ ^47^, ^48^, ^49^ ^]^ at low temperatures that follows a Curie behavior and yields by extrapolation a negative *T_c_* ≈ −70 K^[^ ^50^ ^]^ (see Figure 1c). As a result, already a minute doping of PbTe with GeTe immediately converts Pb_1−_ *_x_* Ge*_x_* Te into a FE material with a critical temperature that rises super linearly with Ge concentration,^[^ ^51^ ^]^ reaching a *T_c_* at room temperature already at *x_Ge_* of about 14% (see Figure 1c), which can be altered by hydrostatic pressure as well.^[^ ^52^ ^]^ At the same time, the direct band gap, low carrier concentration and high mobility is retained.^[^ ^51^, ^53^, ^54^ ^]^

Although the FE distortion in the Pb_1_ *_−x_* Ge*_x_* Te bulk system has been previously demonstrated by means of different techniques like X‐ray and Raman scattering,^[^ ^45^, ^55^ ^]^ capacitance,^[^ ^47^, ^52^ ^]^ transport,^[^ ^51^, ^53^ ^]^ and optical measurements;^[^ ^48^, ^53^, ^54^, ^56^ ^]^ a quantitative assessment on the Rashba spin‐splitting, its correlation with the structural FE distortion, and its evolution with temperature and Ge content still remains elusive. Here, we develop molecular beam epitaxy (MBE) for the growth of high quality Pb_1_ *_−x_* Ge*_x_* Te films in order to realize quantum confined ferroelectric heterostructures and study their structural and electronic properties by combining X‐ray diffraction (XRD) and high‐resolution angle‐resolved photoemission spectroscopy (ARPES). Based on unprecedented ARPES data that we record for artificially designed quantum well (QW) heterostructures, we reveal that Pb_1_ *_−x_* Ge*_x_* Te thin films display extremely sharp quantized subbands that display all key features of a FERSC, namely, that i) they exhibit a giant Rashba effect that is absent in the PE phase and appears below the critical temperature of the FE/PE phase transition, ii) the magnitude of the Rasbha splitting is linear and directly proportional to the FE polarization and precisely follows the temperature dependence of the Landau‐Ginzburg theory of a second order phase transition, iii) the Rashba effect can be controlled and tuned over a wide range by Ge doping and persists down to the ultra‐thin film limit. All taken together, our work originally delivers the complete picture of the unique properties of this novel class of multifunctional materials with great potential for device applications.

## 2 Results and Discussion

### 2.1 Growth

**Figure 2:**
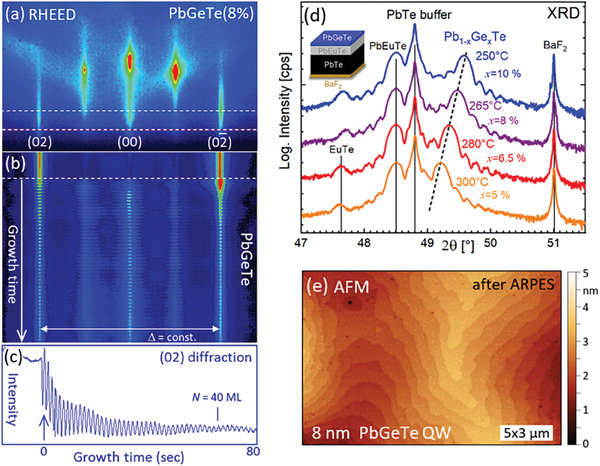
MBE growth and characterization of Pb_1_ *_−x_* Ge*_x_* Te QW heterostructures. a) RHEED patterns and b) evolution of the diffraction intensity profile along the dashed lines in (a) as a function of layer thickness measured in situ during Pb_0.92_Ge_0.08_Te growth on PbTe (111) at a temperature of 265 °C. c) Perfect pseudomorphic 2D growth is signified by the pronounced RHEED intensity oscillations and the constant Δ‐spacing between the diffraction peaks in (b). d) Radial (222) XRD spectra of four 50 nm Pb_1_ *_−x_* Ge*_x_* Te QW layers grown at different temperatures from 250° to 300 °C at the same GeTe/PbTe flux ratio of 1:10. As indicated by the dashed lines, the Pb_1_ *_−x_* Ge*_x_* Te layer peaks indicates a strong increase of the Ge concentration *x_Ge_* when the growth temperature is decreased. The theoretical positions of the BaF_2_, PbTe, Pb_0.9_Eu_0.1_Te, and EuTe peaks (lattice parameters of 6.198, 6.462, 6.492, and 6.598 Å, respectively, see Ref. [62]) are indicated by vertical lines. The finite thickness Laue fringes indicate the high quality of the QW interface. The sample structure is shown as insert. e) AFM surface image of a Pb_0.93_Ge_0.07_Te QW measured after ARPES investigation, revealing a flat surface with only single monoatomic steps and a root mean square roughness below 0.5 nm.

Single crystalline ferroelectric Pb_1_ *_−x_* Ge*_x_* Te films and QW heterostructures were grown by MBE on (111) BaF_2_ substrates^[^ ^62^, ^64^ ^]^ using PbTe and GeTe as source materials (see Experimental Section). In this way, perfect 2D layers were achieved as shown by **Figure** **2**. A key feature of Pb_1_ *_−x_* Ge*_x_* Te growth is the very strong temperature dependence of the Ge incorporation into the epilayers. This is because the vapor pressure of GeTe is three orders of magnitude higher than that of PbTe.^[^ ^65^, ^66^ ^]^ As a result, at the common IV‐VI MBE growth temperatures above 350 °C,^[^ ^62^ ^]^ the re‐evaporation rate of GeTe from the surface exceeds 5 Å s^−1^ so that no GeTe is actually incorporated in the PbTe epilayers.

To overcome this limitation, the substrate temperature for Pb_1_ *_−x_* Ge*_x_* Te has to be drastically reduced below 300 °C to suppress re‐evaporation and achieve a sizeable Ge concentration. This is demonstrated by Figure 2d that shows the (222) XRD spectra of a series of Pb_1_ *_−x_* Ge*_x_* Te epilayers grown on PbTe buffer layers at different temperatures from 250 to 300 °C as indicated. With decreasing growth temperature and a fixed PbTe to GeTe flux ratio of 10:1, one sees that the Pb_1_ *_−x_* Ge*_x_* Te layer peak strongly shifts away from the buffer PbTe peak. This signifies that *x_Ge_* increases from 5% to 10% just by decreasing the substrate temperature from 300 to 250 °C if one considers the change of the Pb_1_ *_−x_* Ge*_x_* Te lattice parameter according to the Vegard's law as:

(1) $$a_{PbGeTe}\;\left(x_{Ge}\right)=\;6.462-0.472\;x_{Ge}\;\left[Å\right]$$

valid for the cubic phase at room temperature.^[^ ^60^, ^67^ ^]^ Using this relation, we find that for a constant GeTe/PbTe flux ratio of 1:10 used for the samples shown in Figure 2d the Ge concentration *x_Ge_* doubles from 5% to 10% when the substrate temperature is decreased from 300 to 250 °C.

For the Pb_1_ *_−x_* Ge*_x_* Te films on PbTe buffer layers shown in Figure 2, however, the lattice of the layer is strained to the that of the buffer layer, which means that the out‐of‐plane lattice parameter is expanded due to the Poisson ratio and thus, the change in the perpendicular direction is amplified by factor of two to Δ*a_z_* (*x_Ge_*) =  0.999Å *x_Ge_* . This is because for *x_Ge_* < 0.15, the thickness of the 50 nm films is below the critical thickness for strain relaxation. This is confirmed by Figure 2b, which shows that the spacing of the (02) streaks of the reflection high energy electron diffraction (RHEED) patterns, recorded during the Pb_1_ *_−x_* Ge*_x_* Te/ PbTe growth, does not change with layer thickness and remains equal to that of the PbTe buffer layer. The perfect 2D growth and the formation of atomically flat surfaces is further witnessed by the pronounced RHEED intensity oscillations shown in Figure 2b,c that persist to more than 40 monolayers. This is corroborated by the atomic force microscopy (AFM) image shown in Figure 2e evidencing that the surface consists of atomically smooth terraces separated only by few single monoatomic steps. The well‐developed finite thickness Laue fringes around the XRD peaks in Figure 2d also demonstrate the high quality of the PbGeTe/PbEuTe interface. Using low temperature growth, we have achieved Pb_1_ *_−x_* Ge*_x_* Te layers with *x_Ge_* up to 0.13, which is well above the Ge solubility limit of ≈0.06 at 300 °C previously determined for bulk material.^[^ ^60^ ^]^

Different layer structures were grown and investigated in this work. Apart from thick films (from 1 to 5 µm) used for the assessment of the FE phase transition by temperature‐dependent XRD measurements, heterostructures consisting of Pb_1_ *_−x_* Ge*_x_* Te QWs with thicknesses down to 8 nm were fabricated by growth on wide band gap Pb_0.9_Eu_0.1_Te barriers (100 nm) pre‐deposited on µm‐thick fully relaxed PbTe buffer and a few nanometers thick EuTe nucleation layer on the BaF_2_ substrate, as shown by the inset of Figure 2d.

Concerning the electrical properties, Ge‐doping slightly increases the tendency of cation vacancy formation that leads to a slight p‐doping of the layers. This is, however, orders of magnitude lower than for GeTe, and thus, this effect can be easily compensated by doping with Bi atoms during growth.^[^ ^44^ ^]^ Bi acts as a donor and leads to an n‐doping. As a result, Pb_1_ *_−x_* Ge*_x_* Te can be made p‐ or n‐ type depending on the extrinsic Bi‐doping. Accordingly, in our thick samples and our heterostructures, carrier densities from 10^17^ to 10^19^ cm^−3^ n‐ or p‐type were obtained. The carrier mobility is found as high as 1 000 cm^2^ V^−1^ s^−1^ for low Ge content samples (*x*_Ge_ ≲ 4%) at room temperature, and decreases to several hundred of cm^2^ V^−1^ s^−1^ for higher Ge content. As a result, Pb_1_ *_−x_* Ge*_x_* Te displays superior transport properties compared to GeTe.^[^ ^51^, ^53^ ^]^

### 2.2 Ferroelectric Phase Transitions and Sublattice Displacement

**Figure 3:**
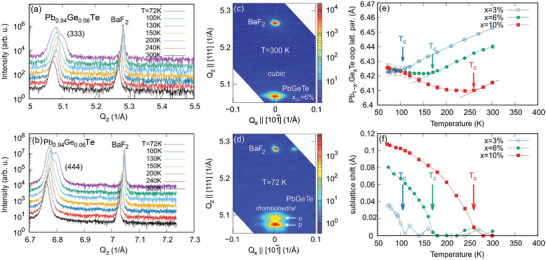
Ferroelectric structural phase transition in Pb_1_ *_−x_* Ge*_x_* Te. a,b) XRD scans across the (333) and (444) Bragg reflections measured at various temperatures for Pb_0.94_Ge_0.06_Te on BaF_2_ (111). c,d) RSMs around the (333) reciprocal lattice point at 300 and 72 K, respectively. e) Temperature dependence of the out‐of‐plane lattice parameter of Pb_1_ *_−x_* Ge*_x_* Te with 0 < *x* < 0.1, showing the elongation of the unit cell along the [111] direction occurring below the critical temperature *T_c_*. f) Same for the measured anion/cation sublattice shift δ*u* along the [111] direction determined from the change of the intensity ratio between (333) and (444) diffraction peaks. The critical temperatures are indicated by the arrows.

To assess the FE phase transition, temperature dependent XRD measurements were performed on a series of Pb_1_ *_−x_* Ge*_x_* Te films with 0 < *x_Ge_* < 0.13. The results are presented in **Figure** **3**, where in panels (a,b) radial XRD scans across the (333) and (444) Bragg reflections for *x_Ge_* =  0.06 are depicted. Evidently, with decreasing temperature, the Pb_0.96_Ge_0.06_Te diffraction peak is seen at first to shift parallel to the BaF_2_ substrate due to the shrinking of the lattice parameter by the ordinary thermal contraction of the materials; however, at a critical temperature of *T_c_* =  160 K the peaks shift suddenly reverses its direction, signifying the onset of the FE phase transition in which the unit cell becomes rhombohedrally distorted, that is, elongated along the [111] direction (see Figure 1a) and the rhombohedral lattice angle decreases as shown in the Figure S1, Supporting Information. In addition, below *T_c_* the Pb_0.96_Ge_0.06_Te peak splits up into two peaks clearly signifying the symmetry reduction from the cubic to the rhombohedral phase, where the different <111> directions of the crystal lattice are no longer equivalent and FE domains with different elongation directions are formed. While in bulk Pb_1_ *_−x_* Ge*_x_* Te these domains simultaneously appear with equal ratio, in our epitaxial (111) oriented films the “*p*”‐domains with the rhombohedral elongation along the [111] surface normal is favored over the “*o*”‐ domains, where the elongation is along one of the three $\left\langle \bar{1}11\right\rangle$ distortions (oblique to the surface). Accordingly, in the diffraction patterns the “*o*”‐ domains appear only as a smaller shoulder on the right hand side of the main peak in Figure 3a,b. This is corroborated by the (333) reciprocal space maps (RSMs) displayed in Figure 3c,d showing that above *T_c_* only a single Pb_1_ *_−x_* Ge*_x_* Te diffraction peak appears, whereas below *T_c_* two well‐separated peaks are observed, one for the “*p*” domain and one for the “*o*” domains. This splitting is a key indication for the symmetry reduction caused by the structural phase transition. Moreover, below *T_c_* the magnitude of the splitting strongly increases with decreasing temperature (see Figure 3a,b) because the rhombohedral distortion increases with decreasing temperature.

The rhombohedral distortion of the unit cell also changes the lattice parameter as signified by Figure 3e, where the measured out‐of‐plane lattice parameters *a*_111_ of the Pb_1_ *_−x_* Ge*_x_* Te layers is plotted as a function of temperature for samples with different compositions. As indicated by the arrows, the slope of *a*_111_(*T*) clearly changes sign at the critical temperature, that marks the onset of the cubic‐to‐ rhombohedral phase transition. This is because in the FE phase the lattice gets elongated along the [111] direction and thus, the out‐of‐plane lattice parameter increases. From the onset of this slope change, the critical temperatures *T_c_* were derived, increasing from 100 to 160 and 260 K for *x_Ge_* = 0.03, 0.06, and 0.1, respectively. As shown by Figure 1c this dependence can be described by the relation $T_{c}\;\left(x_{Ge}\right)=\;1000\sqrt{x_{Ge}}-70$ [K], which is in good agreement with the literature (see Figure 1c) where the *T_c_* has been obtained by a large variety of experimental techniques such as EXAFS,^[^ ^55^ ^]^ capacitance,^[^ ^47^, ^50^, ^52^, ^58^ ^]^ transport^[^ ^51^, ^53^ ^]^ and specific heat^[^ ^68^ ^]^ measurements, Raman^[^ ^45^, ^46^, ^61^ ^]^ and optical^[^ ^48^, ^53^, ^54^, ^56^ ^]^ spectroscopies.

It is noted, however, that the rhombohedral distortion alone is not sufficient evidence for the existence of a FE state. For a FE polarization to emerge, the lattice centrosymmetry must be broken in addition, and the anion/cation sublattices must be shifted with respect to each other, as shown schematically in Figure 1a. To assess and quantify this shift, we analyze the intensity evolution of the (*hkl*) Bragg peaks as a function of temperature. This quantity will reveal the change of the structure factor |*F_hkl_* | occurring when atom positions are shifted within the unit cell at the FE phase transition. This intensity change is particularly pronounced for *odd* (*hkl*) reflections where the waves scattered by the anions and cations are exactly out of phase in the centrosymmetric $Fm\bar{3}m$ cubic structure. Thus, even minute changes in the anion/cation lattice position due to the transition to the non‐centrosymmetric *R*3*m* phase yield a relatively large change in diffraction intensity of the odd (*hkl*) reflections, whereas for even (*hkl*) the change is negligible because the scattered waves are in phase. This is exactly what we observe for the Pb_1_ *_−x_* Ge*_x_* Te (333) and (444) Bragg peaks as shown in Figure 3a,b, where below *T_c_*, the (333) intensity rises rapidly by more than a factor of two, whereas the (444) Bragg peak intensity is essentially constant (see Figure S1, Supporting Information).

Because the sublattice shift δ*u* in Pb_1_ *_−x_* Ge*_x_* Te is along the [111] direction, δ*u* can be directly determined from the intensity of the odd (*hkl*) of the FE phase relative to the cubic PE phase according to:^[^ ^41^ ^]^

(2) $$\frac{I_{hkl}^{FE}}{I_{hkl}^{PE}}=cos^{2}\;\left\{2\pi \left(h+k+l\right)\delta \right\}+{\left(\frac{f_{A}+f_{C}}{f_{A}-f_{C}}\right)}^{2}sin^{2}\left\{2\pi \left(h+k+l\right)\delta \right\}$$

where *f_A_* and *f_C_* are the anion and cation form factors and δ denotes the normalized deviation of the cation/anion lattice planes from the center position given by $\delta u\;=\;2a\sqrt{3}\delta$. From this analysis, we thus quantitatively obtain the dependence of δ*u* as a function of *x_Ge_* and temperature. As shown by Figure 3f this directly evidences that the onset of the PE‐FE phase transition is perfectly correlated with the appearance of a sublattice shift at exactly the same critical temperature. Below *T_c_*, the sublattice shift δ*u* increases and approaches a low temperature saturation value that linearly scales with the Ge content, reaching a value of δ*u* ≈ 0.11 Å for *x_Ge_* = 0.1 at T = 72 K. This corresponds to a change in the anion/cation lattice plane spacing as large as 3%, that is about half of the GeTe value.^[^ ^30^, ^33^ ^]^

### 2.3 Ferroelectric Rashba Effect

**Figure 4:**
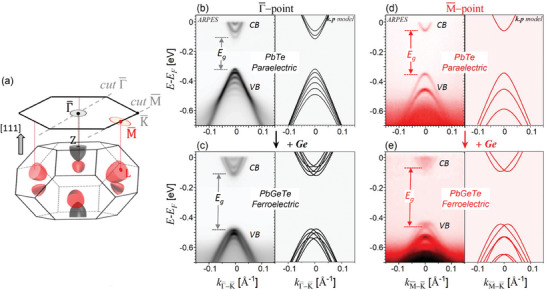
Ferroelectricity and Rashba effect induced by Ge doping. a) 3D BZ of the Pb_1_ *_−x_* Ge*_x_* Te rhombohedral lattice with an elongation along [111] as well as the schematic Fermi pockets in red and black. The 2D projection of the BZ and of the Fermi pockets on the (111) surface is illustrated along with the ARPES measurement directions. b,c) ARPES measurements at *T* = 10 K along the $\bar{K\Gamma K}$ direction for the PbTe (b) and the Pb_0.93_Ge_0.07_Te (c) 9 nm QWs. The fits using the ***k***. ***p*** model developed in this work are plotted on the right of the corresponding ARPES spectra. Note that in order to better resolve the confined states of the conduction band and QW gap, the contrast of the CB in the ARPES spectra has been enhanced by a factor of two. d,e) Similar than (b,c) along the $\bar{\mathrm{KMK}}$ direction.

ARPES was employed to resolve the impact of ferroelectricity on the electronic band structure. To this end, we have prepared Pb_1_ *_−x_* Ge*_x_* Te heterostructures consisting of 9 nm‐thick QWs to obtain single domain films in which the formation of “*o*”‐domains is completely suppressed. These QWs were grown on top of 100 nm thick, wide band gap Pb_0.9_Eu_0.1_Te barrier layers in order to effectively confine the electrons and holes in the FE Pb_1_ *_−x_* Ge*_x_* Te layer, which, as shown in **Figure** **4**, enhances the signature of the Rashba effect. ARPES measurements were performed at the high symmetry points $\bar{\Gamma }$ and $\bar{M}$ of the 2D Brillouin zone (BZ) where the band extrema are located, as represented in Figure 4a.

ARPES measurements of PbTe and Pb_0.93_Ge_0.07_Te QWs around the $\bar{\Gamma }$ and $\bar{M}$‐points are shown in Figure 4b–e together with their fit using ***k***. ***p*** theory as derived below for cubic and rhombohedral lattices (see the Experimental Section). Due to the quantization of the electronic states in the QW, a large number of quantum confined states appear in the ARPES spectra both for the PbTe as well as the Pb_0.93_Ge_0.07_Te QW with sharp subband dispersions (<20 meV line width) that are perfectly reproduced by the ***k***. ***p*** calculations with the parameters given in the Supporting Information. Evidently, the quantized subbands of Pb_0.93_Ge_0.07_Te QW are split in the *k*_∥_‐direction. This is a clear indication for the FE Rashba effect because this phenomenon is obviously absent in the paraelectric PbTe QW case (Figure 4b,c).

For the $\bar{\Gamma }$‐point, the energy level spacing between the quantum confined states is found to be significantly smaller than for the $\bar{M}$‐points. This is due to the large difference between the energy‐momentum dispersions of the two types of valleys in the quantization direction resulting from the large effective mass anisotropy in this system (see the schematic ellipsoidal Fermi surfaces in Figure 4a).^[^ ^69^, ^71^ ^]^ This corresponds to an about ninetimes heavier confinement mass at the $\bar{\Gamma }$‐point, which makes the quantum confinement weaker than at $\bar{M}$. At the $\bar{\Gamma }$‐point (Figure 4c), the Rashba splitting of the Pb_0.93_Ge_0.07_Te QW is well‐resolved at high momenta, but close to $\bar{\Gamma }$ there is a strong overlap of the individual subbands. This is well‐reproduced by the ***k***. ***p*** calculations displayed on the right‐hand side of Figure 4c. For this reason, the individual subband dispersions are difficult to distinguish by ARPES. At the $\bar{M}$‐point (Figure 4e), this strong overlap is absent because of the large energy splitting of the energy levels and thus, the Rashba spin‐splitting induced by Ge doping is well‐resolved. This facilitates the direct comparison with the ***k***. ***p*** calculations including the Rashba effect and evidences a perfect agreement between theory and experiments. Altogether, the dispersions measured at both valleys unequivocally demonstrates the induction of a Rashba spin splitting by Ge doping and thus, that the Pb_1_ *_−x_* Ge*_x_* Te is a ferroelectric Rashba semiconductor.

**Figure 5:**
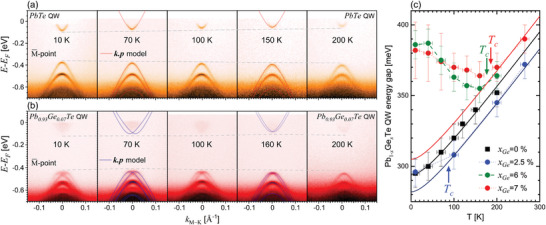
Temperature dependence of the Rashba effect. a,b) ARPES spectra of PbTe (orange) and Pb_0.93_Ge_0.07_Te (red) 9 nm‐thick QWs at different temperatures from 10 to 200 K. The red and blue lines denote the fit using the ***k***. ***p*** model shown for *T* = 70, *T* = 150, and *T* = 160 K. c) QW band gaps versus temperature of the four investigated ARPES samples. The green (blue) data points correspond to a Pb_0.94_Ge_0.06_Te (Pb_0.975_Ge_0.025_Te) QW shown in the Supporting Information. The dashed lines are guide‐for‐the‐eyes and the solid lines represent the expected gap dependence in the cubic phase varying like 0.5*T* ^2^/(*T* + 55) meV with temperature.^[^ ^56^ ^]^ The critical temperatures are indicated.

To further characterize the FERSC of Pb_1_ *_−x_* Ge*_x_* Te, we have measured the temperature dependence of the Rashba splitting, focusing on the ARPES dispersions at the $\bar{M}$‐points, where the quantized subbands are well resolved (see Figure 4d,e). The results are shown in **Figure** **5**a,b where the ARPES maps recorded at 10 < *T* < 200 K are compared for the PbTe and the Pb_0.93_Ge_0.07_Te QWs, respectively. Evidently, no Rashba splitting is observed for the PbTe QW at all temperatures. The electronic subbands remain spin degenerate, meaning that neither a FE polarization nor any surface band bending is present in this sample. This is fully coherent with the fact that PbTe crystallizes in a cubic lattice and no rhombohedral distortion emerges at any temperature. Therefore, PbTe stands as a reference cubic phase persisting down to low temperature.

For the Pb_0.93_Ge_0.07_Te QW, the Rashba spin‐splitting is found to gradually decrease with increasing temperature. At *T*  =  200 K and above, only single sharp bands are observed. The Kramers degeneracy is lifted only below *T_c_* ≈ 190 K where the system is in the FE phase. As no external nor internal magnetism, nor an external electric field is present in our experiments, this is an unambiguous evidence for the FE origin of the Rashba effect. We can also safely rule out the presence of a surface‐induced Rashba effect caused, for example, by the presence of an accumulation or a depletion layer at the surface because this would result in a temperature *independent* Rashba spin‐splitting. Because it appears only below a certain *T_c_*, the Rashba effect we observe here is completely distinct from the conventional surface Rashba splitting induced by surface band bending potentials caused by surface doping, Fermi level pinning or adatom adsorption.^[^ ^44^, ^72^, ^73^ ^]^ In such a case, the Rashba splitting should not show a temperature dependence perfectly correlated to the FE transition. Moreover, no Rashba splitting is observed for the PE cubic phase as well as the undoped PbTe reference layer (see Figure 5a), confirming the absence of surface band bending. Therefore, our measurements show that the Rashba spin splitting emerging below *T_c_* is exclusively due to the intrinsic electric field caused by the FE polarization of the rhombohedral phase. The temperature dependent Rashba splitting is a second key signature of the FE phase transition occurring in Pb_1_ *_−x_* Ge*_x_* Te. Similar data was obtained for additional Pb_1_ *_−x_* Ge*_x_* Te samples with different Ge concentrations, as shown in the Supporting Information.

The FE phase transition in Pb_1_ *_−x_* Ge*_x_* Te also induces an anomalous temperature dependence of the energy gap as shown in Figure 5c. This gap was extracted from the ARPES spectra of the Pb_1_ *_−x_* Ge*_x_* Te QWs by measuring the energy separation between the electron and hole ground confined states at *k*_‖_ = 0 by fitting the dispersions by ***k***. ***p*** theory as shown by the solid lines in Figure 5a,b. We clearly see that at the critical temperature *T_c_* at which the Rashba splitting appears, the slope of the gap versus temperature shows a clear sign change of its slope and increases at low temperatures.^[^ ^53^, ^56^ ^]^ This is in complete contrast to the monotonic decrease of the band gap of PbTe (Figure 5a) with decreasing temperature that is in perfect agreement with the literature.^[^ ^56^, ^74^ ^]^ The same effect is observed for the quantum confined states at the $\bar{\Gamma }$‐point of the surface Brillouin zone as shown by the first derivative spectra in Figure S5, Supporting Information. Accordingly, this gap anomaly of Pb_1_ *_−x_* Ge*_x_* Te is a third clear signature of the FE phase transition, its origin is discussed in the theory part detailed in the Experimental Section. Taken all together, our experimental data evidences the simultaneous occurrence of the FE transition, Rashba effect and gap anomaly in thin film Pb_1_ *_−x_* Ge*_x_* Te heterostructures at exactly the same critical temperature. Most importantly, repeated measurements during several temperature cycles show that the gap anomaly and the Rashba splitting emergence is fully reproducible and completely reversible with temperature as expected for a FE phase transition.

In order to get more quantitative insight on the FE Rashba effect and its interplay with the FE lattice distortion, we have modelled the ARPES data with a refined ***k***. ***p*** model (Ref. [54]) extended to describe the electronic structure of QWs in their rhombohedral phase. The modelling was done by decomposing the FE distortion into a strain effect and a relative shift of the anions/cation sublattices (see Experimental Section). The strains caused by the rhombohedral distortion are taken into account by diagonal matrix elements in the ***k***. ***p*** Hamiltonian that enlarge the energy gap. On the other hands, the anion/cation sublattice shift responsible for the electric dipole is considered by adding two interband coupling parameters that appear as *k*‐linear terms in the ***k***. ***p*** Hamiltonian and account for the well‐known Rashba parameter, α_*R*_, which governs the band splitting observed in ARPES.

**Figure 6:**
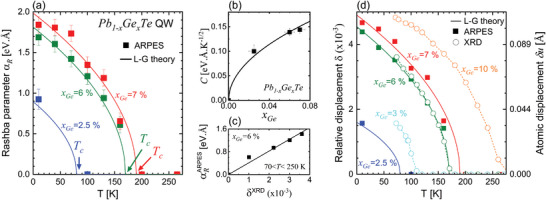
Interplay between structural and electronic properties of a FERSC. a) Rashba parameters of Pb_1_ *_−x_* Ge*_x_* Te versus temperature determined by ARPES. The solid lines correspond to the $\alpha_{R}\;\left(T\right)=\;C\sqrt{T_{C}-T}$ dependence expected from the Landau‐Ginzburg theory (“L‐G theory”) for a second‐order phase transition. b) Plot of the coefficient *C* determined in (a) versus the Ge concentration (dots). The solid line shows the experimental fit using $C\;\left(x_{Ge}\right)=\;0.57\sqrt{x_{Ge}}$. c) Rashba parameters determined in ARPES between 70 and 250 K for the Pb_0.94_Ge_0.06_Te QW (green dots in (a)) as a function of the relative atomic displacement measured in XRD (see Figure 3f). The solid line is computed using Equation (3) with Ξ_*o*_ =  16.5 eV. d) Atomic relative displacement δ as a function of temperature. The square dots are the value deduced from the Rashba constant determined by ARPES, and the circles represent the values measured in XRD (see Figure 3f). Dashed lines are guide‐for‐the‐eyes. Solid lines represent the $\sqrt{T_{C}-T}$ dependence deduced from (a) using Equation (3) with Ξ_*o*_ =  16.5 eV. The sublattice shift $\delta u\;=\;2\delta \sqrt{3}a$ that is shown on the right axis is calculated using a temperature constant lattice parameter *a*  =  6.425 Å.

Calculations are described in the Experimental Section and with appropriate choice of parameters they precisely agree with ARPES spectra. This is demonstrated by the solid lines in Figure 5 that represent the calculated QW band dispersions for selected temperatures of 70 and 150K. It is emphasized that the fit of the calculations to ARPES is excellent for PbTe and Pb_1_ *_−x_* Ge*_x_* Te both in their respective cubic and rhombohedral phases. In particular, for the FE phase the calculations involve the additional *k*‐linear Rashba terms that reproduce the observed Rashba spin splitting of the subbands, while these terms are absent in the cubic phase. The Rashba parameter α_*R*_ obtained by the fit of the experiments is plotted in **Figure** **6**a versus temperature for three samples with different *x_Ge_*. In all cases, the Rashba parameter is found to rapidly increase when temperature decreases below *T_C_* and to be zero for *T* > *T_C_*.

The Rashba parameter, which is responsible for the spin‐splitting of the bands, is directly related to the FE polarization via the optical deformation potential Ξ_*o*_ according to:^[^ ^54^ ^]^

(3) $$\alpha_{R}=\frac{\hbar v\Xi_{o}\sqrt{3}}{E_{g}}\;\delta \;=\frac{\hbar v\Xi_{o}}{2aqNE_{g}}\;P_{elec}$$

where *E_g_* is the energy gap of the QWs, *v* is the Dirac velocity and δ is the off‐center lattice shift of the anions/cations in units of the lattice parameter *a* ($\delta u\;=\;2a\sqrt{3}\delta$) as already defined in the previous section. The polarization can be written as $P_{elec}=\;Nq\delta u\;=\;2aqN\sqrt{3}\delta$, where *q* is the charge of the dipole moment and *N* the number of dipoles per volume unit. The key result here is that the Rashba parameter is directly proportional to the sublattice shift, thus, the FE polarization.

In the Landau‐Ginzburg theory, the polarization stands as the order parameter defining the FE phase transition. Within the framework of this theory, one gets $P_{elec}\propto \sqrt{T_{C}-T}$ in the FE phase^[^ ^75^, ^76^ ^]^ and thus, $\alpha_{R}\propto \sqrt{T_{C}-T}$ according to Equation (3), neglecting the small temperature variation of *E_g_*, *a* and *v*. As shown by the solid lines in Figure 6a this dependence is fully verified by the perfect fit of $\alpha_{R}=\;C\sqrt{T_{C}-T}$ to the experimental data as predicted by the Landau‐Ginzburg theory. The fit yields an accurate determination of the coefficient *C*, whose dependence on Ge concentration is shown in Figure 6b and is phenomenologically described by $C\;\left(x_{Ge}\right)=\;0.57\sqrt{x_{Ge}}$ in unit of eV Å K^−1/2^ within the investigated range of 0 < *x_Ge_* < 0.07. These results imply that the FE phase transition in our Pb_1_ *_−x_* Ge*_x_* Te QW heterostructures is very well‐described by a second order phase transition. At low temperature, the Rashba constant reaches an experimental value of α_*R*_ =  2 eV Å in the Pb_0.93_Ge_0.07_Te QW heterostructure (see Figure 6a). This value of α_*R*_ has been experimentally determined by fitting the ARPES spectra using the ***k***. ***p*** theory developed in this work (see Figure 5b and Experimental Section). It is comparable to state‐of‐the‐art Rashba systems like BiTeI ( α_*R*_ =  3.8 eV Å),^[^ ^35^ ^]^ SnTe ( α_*R*_ =  4.4 eV Å),^[^ ^6^ ^]^ Bi‐doped Pb_1_ *_−x_* Sn_x_Te ( α_*R*_ =  2 − 4 eV Å)^[^ ^44^ ^]^ or even GeTe ( α_*R*_ =  4.2 − 4.8 eV Å),^[^ ^40^ ^]^ indicating a giant Rashba effect in Pb_1_ *_−x_* Ge*_x_* Te already at small Ge concentrations.

Most importantly, based on our XRD and ARPES data, we demonstrate for the first time the direct quantitative relation between the FE polarization – the sublattice shift – and the electronic spin texture – the Rashba spin splitting – in a FERSC. This is clearly evidenced by Figure 6c, where the Rashba parameter α_*R*_ determined by ARPES is plotted as a function of the relative atomic displacement δ measured by XRD for Pb_0.94_Ge_0.06_Te. The linear relation between these two quantities (see Equation (3)) is fully confirmed and yields the optical deformation potential Ξ_*o*_ =  16.5 eV, in good agreement with previous literature.^[^ ^53^, ^54^ ^]^

In order to illustrate the unique structure‐electronic correlation of FERSC, Figure 6d shows the temperature evolution of δ measured by XRD, as well as the δ deduced from the Rashba constant α_*R*_ measured by ARPES using Equation (3). The best fit between ARPES and XRD data directly yields the optical deformation potential Ξ_*o*_ =  16.5 eV for the all range of investigated Pb_1_ *_−x_* Ge*_x_* Te. This directly demonstrates that the sublattice shift in a FERSC is the key source responsible for the Rashba splitting, and that the displacement strength controls the Rashba constant. These results show the equivalence between structural and electronic properties, that is, between the FE polarization strength and Rashba spin splitting, in FERSC.

## 3 Conclusion

In summary, we have described the MBE growth and characterized the structural and electronic properties of ferroelectric single crystalline epitaxial Pb_1_ *_−x_* Ge*_x_* Te layers over a large range of temperature and Ge contents, qualifying this material as a FERSC with outstanding properties sustained even in the thin film limit. The ferroelectric structural phase transition was revealed by temperature dependent XRD experiments, showing that the sublattice shift responsible for the non‐centrosymmetry reaches values exceeding 0.1 Å already at *x_Ge_* ≈ 10%, which leads to a giant Rashba effect observed by ARPES with an experimentally determined Rashba coupling constant as large as 2 eV Å. The temperature dependent Rashba splitting precisely follows the behavior predicted by the Landau‐Ginzburg theory of a second‐order phase transition. Furthermore, the magnitude of the Rashba effect is shown to be linearly proportional to the anion versus cation atomic displacement, and thus, to the electric polarization, which is the key signature of its ferroelectric origin.

In this way, Pb_1_ *_−x_* Ge*_x_* Te stands for a highly promising FERSC system because it features a number of advantages over other FERSC materials, namely, i) a low doping level, ii) a direct optical gap, iii) a tunable and high critical temperature due to the ternary nature of the Pb_1_ *_−x_* Ge*_x_* Te alloy, and iv) a high Rashba spin‐splitting in nanometric layers. This opens up new avenues for the realization of FERSC devices dedicated to a large number of applications.

## 4 Experimental Section

### Growth

MBE growth of Pb_1_ *_−x_* Ge*_x_* Te layers and QWs on BaF_2_ (111) substrates was performed using a VARIAN Gen II MBE system under ultra‐high vacuum (UHV) conditions (2 × 10^−10^ mbar) using PbTe, GeTe and Bi_2_Te_3_ as source materials. The composition of the ternary layers Pb_1_ *_−x_* Ge*_x_* Te was controlled by the GeTe/PbTe beam flux ratio measured precisely using a quartz microbalance moved into the substrate position and the sample temperature was measured with an infrared pyrometer. The growth rates were set to about 0.3 nm s^−1^ and the GeTe/PbTe beam flux ratio varied in the range from 1:5 to 1:20. The growth was monitored in‐situ using RHEED and the sample surface characterized by AFM using a Veeco Dimensions 3100 SPM. Bulk‐like films with 4 – 5 µm thickness with different composition up to x_Ge_ = 0.13 were grown for the temperature dependent XRD studies to assess the FE phase transitions. For ARPES, PbTe and Pb_1_ *_−x_* Ge*_x_* Te QW of 8–10 nm thickness were grown on 100 nm thick wide band gap Pb_0.9_Eu_0.1_Te barrier layers and a PbTe/EuTe buffer layer in order to achieve a quantum confinement of the electronic states in the Pb_1_ *_−x_* Ge*_x_* Te layers. These samples were intentionally n‐doped using in‐situ Bi doping in order to bring the Fermi level sufficiently high into the conduction band to allow the observation of both hole and electron states by ARPES.

### Structure Characterization and FE Phase Transition

The sample structure was characterized in detail to determine the lattice parameter, thickness and layer composition using a standard Pananalytical materials research diffractometer as well as a Rigaku SmartLab X‐ray diffractometer equipped with a custom‐made variable temperature sample stage with hemispherical PEEK window (ColdEdge International) for temperature dependent measurements down to 72 K using pumped liquid nitrogen. Both instruments were equipped with a Cu X‐ray source and a Ge(220) channel‐cut monochromator. The in‐plane and out‐of‐plane lattice parameters as well as the FE lattice distortion was determined from RSMs recorded around the (333), (444), and (531) reflections.

### Angle‐Resolved Photoemission Spectroscopy

ARPES measurements were performed at the high‐resolution URANOS beamline at the SOLARIS synchrotron in Krakow, Poland. For this purpose, the samples were transferred from the MBE to the synchrotron under UHV conditions using a battery‐operated Ferrovac vacuum suitcase. UV radiation of 18 eV was used for excitation of the photoelectrons and their angular and energy distribution measured by a VG Scienta DA30L electron spectrometer with energy and angular resolution better than 3 meV and 0.1°, respectively. Temperature‐dependent measurements were taken under UHV conditions (<10^−10^ mbar) with a horizontally polarized light and a vertical slit.

### Modelling of the Band Structure in FERSC Heterostructures using ***k***. ***p*** Theory

The ***k***. ***p*** Hamiltonian to model the Pb_1_ *_−x_* Ge*_x_* Te QW heterostructures was modified from the Hamiltonian given in Ref. [54] for rhombohedral bulk Pb_1_ *_−x_* Ge*_x_* Te by including the z‐dependent quantum confinement potential. It writes:

(4) $$H\;=\left(\begin{array}{cccc} -V\left(z\right)+\alpha_{2,∥}k_{x} & -i\alpha_{1,∥}k_{-}-i\alpha_{2,z}\frac{d}{dz} & -i\hbar v_{z}\frac{d}{dz} & \left(u^{2}-v_{1}^{2}\right)v_{∥}\hbar k_{-}\\ i\alpha_{1,∥}k_{+}-i\alpha_{2,z}\frac{d}{dz} & -V\left(z\right)-\alpha_{2,∥}k_{x} & \left(u^{2}-v_{1}^{2}\right)v_{∥}\hbar k_{+} & i\hbar v_{z}\frac{d}{dz}\\ -i\hbar v_{z}\frac{d}{dz} & \left(u^{2}-v_{1}^{2}\right)v_{∥}\hbar k_{-} & V\left(z\right)+\alpha_{2,∥}k_{x} & -i\alpha_{1,∥}k_{-}-i\alpha_{2,z}\frac{d}{dz}\\ \left(u^{2}-v_{1}^{2}\right)v_{∥}\hbar k_{+} & i\hbar v_{z}\frac{d}{dz} & i\alpha_{1,∥}k_{+}-i\alpha_{2,z}\frac{d}{dz} & V\left(z\right)-\alpha_{2,∥}k_{x} \end{array}\right)\;$$

Here, *v_z_* and *v*_∥_ are the out‐of‐plane and in‐plane Dirac velocities accounting for the anisotropy; *k*_±_ = *k_x_*  ± *ik_y_*; *V*(*z*) is the confinement potential that is *E_g_*/2 in the PbGeTe quantum well, 450 meV in the Pb_0.9_Eu_0.1_Te barrier underneath, and is taken as 2 eV to mimic the vacuum barrier at the surface. The terms which appear with the FE distortion are set as:

(5) $$\alpha_{1,2,∥,z}=\;2uv_{1,2}\hbar v_{∥,z}$$

where *u* and *v*_1,2_, introduced in Ref. [54] parametrize the lattice distortion. Indeed, *u*  =  1 denotes the cubic phase (with *v*_1_ =  0 and *v*_2_ =  0), and the ferroelectric polarization increases as *u* moves away from unity to lower values. They are given by:

(6) $$\begin{array}{c} \left\{\begin{array}{c} u\;=\cos\theta \;\\ \;v_{1}=\sin\theta \;\cos\phi \\ \;v_{2}=\sin\theta \;\sin\phi \end{array}\right.\; \end{array}$$

So that $u^{2}+v_{1}^{2}+\;v_{2}^{2}=\;1$. Here, the angles θ and ϕ are defined in terms of matrix elements Δ_1_ and Δ_2_, which couple orbitals and orbitals and spins, respectively.

(7) $$\begin{array}{c} \left\{\begin{array}{c} \;\tan\phi =\frac{\Delta_{2}}{\Delta_{1}}\;\\ \;\tan2\theta =\frac{1}{\sqrt{\frac{E_{g}^{2}}{4\left(\Delta_{1}^{2}+\Delta_{2}^{2}\right)}-1}}\; \end{array}\right. \end{array}$$

Additional matrix elements were included to take into account the dilatation and shear strains emerging in the rhombohedral phase γ  = δ_1_  − δ_2_ following the notation used in Ref. [54] They emerged as diagonal terms in the Hamiltonian and were taken into account in the energy gap parameter *E_g_*. Consequently, the PbGeTe energy gap was renormalized to:

(8) $$E_{g}=\sqrt{{\left(2\Delta +\gamma \right)}^{2}+4\left(\Delta_{1}^{2}+\Delta_{2}^{2}\right)}\;$$

where 2Δ is the energy gap if the lattice was in the cubic phase. This explains the anomalous temperature dependence of the energy gap observed in Figure 5c. However, the lack of independent information on γ and Δ_2_ (see below) prevents us from drawing any further conclusions.

In summary, the rhombohedral distortion introduces the four following parameters.

- Intraband matrix elements δ_1,2_ that account for the strains.
- Interband matrix elements Δ_1,2_ accounting for the electric dipole, emerging from the sublattice shift.

For further details, the reader is referred to Ref. [54] and to equivalent theories developed in Refs. [53, 77]

To find the confined state dispersions of the FERSC quantum well, the problem is first solved exactly considering the following Hamiltonian:

(9) $$H_{0}=\left(\begin{array}{cccc} -V\left(z\right) & 0 & -i\hbar v_{z}\frac{d}{dz} & 0\\ 0 & -V\left(z\right) & 0 & i\hbar v_{z}\frac{d}{dz}\\ -i\hbar v_{z}\frac{d}{dz} & 0 & V\left(z\right) & 0\\ 0 & i\hbar v_{z}\frac{d}{dz} & 0 & V\left(z\right) \end{array}\right)\;$$

This was performed in Ref. [78, 79]. One obtains the energy of the confined states as well as their wavefunctions ψ_*n*_. The additional terms in *k_x_*, *k_y_* and α_2,*z*_ were taken into account in a perturbation theory. The matrix 〈ψ_*n*_|δ*H*|ψ_*m*_〉 with *n* and *m* denoting the confined states, and δ*H*  =  *H* − *H*_0_ was then solved numerically and gives the *k*‐dispersions of the confined states. Moreover, for a thin layer, the ferroelectric distortion is likely to occur in the direction perpendicular to the surface, and thus, leads to one single domain. This was demonstrated by the XRD measurements performed on thick films and detailed in Figure 3b,c, showing that the prominent domain is the one with the elongation perpendicular to the surface. In this way, one gets Δ_2_ =  0 at the $\bar{\Gamma }$‐point by symmetry consideration.^[^ ^54^, ^77^ ^]^ More generally, the matrix elements γ, Δ_1_ and Δ_2_ have no reason to be equal at the $\bar{\Gamma }$ and the $\bar{M}$‐points.

*At the* $\bar{\Gamma }$ *‐point*, the results are analytical under a good approximation. The *n* ^th^ subband dispersions at the $\bar{\Gamma }$‐point for Pb_1_ *_−x_* Ge*_x_* Te in its rhombohedral phase write:

(10) $$\begin{array}{c} \;E_{n}=\;\pm \sqrt{\beta_{n}^{2}+\hbar^{2}v^{2}k^{2}\pm 2\alpha_{R}\beta_{n}\left|k\right|} \end{array}$$

Here,β_*n*_ is the energy of the *n* ^th^ subband at *k*  =  0 obtained by solving *H*_0_, and α_*R*_ the Rashba constant. Equation (10) stands as the dispersion of Rashba‐split Dirac bands. Note that the dispersion is isotropic at the $\bar{\Gamma }$‐point. If *E_R_* is defined as usual as the energy difference between the top of the valence band and the energy at *k*  =  0; and *k_R_* as the position of the valence band maximum,^[^ ^4^, ^80^ ^]^ then the Rashba parameter α_*R*_ writes:

(11) $$E_{R}=\beta_{n}\;\left[1-\sqrt{1-\frac{\alpha_{R}k_{R}}{\beta_{n}}}\right]\Leftrightarrow \;\alpha_{R}=\frac{2E_{R}}{k_{R}}\;\left[1-\frac{E_{R}}{2\beta_{n}}\right]$$

This formula was derived in the framework of a 2‐band Dirac model, thus, well‐adapted for narrow gap materials. For relatively wide gap materials, Equation (11) is well approximated by the formula obtained with a one band parabolic model.^[^ ^4^, ^80^ ^]^ The factor 1 − *E_R_*/(2β_*n*_) stems from the Dirac nature of the material. Note that at high momenta, Equation (10) for the valence states tends toward:

(12) $$E_{n}=\;-\hbar \mathrm{vk}\pm \frac{\alpha_{R}\beta_{n}}{\hbar v}$$

Thus, at a fixed high momentum, the spins were shifted by 2α_*R*_β_*n*_/ℏ*v* in energy, which gives an experimental estimation of the Rashba constant α_*R*_ at the $\bar{\Gamma }$‐point. For the Pb_0.93_Ge_0.07_Te QW at low temperature (see Figure 4c), one finds α_*R*_ ≈ 0.8 eV Å. This gave a relatively small value of Δ_1_ ≈ 12 meV and thus, a non‐negligible γ value to explain the gap anomaly observed in Figure 5c and in the Supporting Information (see Equation (8)).

*At the* $\bar{M}$ *‐points*, one needed to rotate *H* to align the *z* axis with the great axis of the oblique valley, which was tilted by 70.5°. Such a rotation is described in Ref. [79] and prevents us from using analytical expressions. Moreover, the ARPES measurements were taken along the $\bar{M}-\bar{K}$ direction (see Figure 4a), which corresponds to *k_y_* in the coordinates. Therefore, the dispersions were calculated for *k_x_* =  0, which means that the parameter Δ_2_ (or *v*_2_) does not intervene in the fit of the measured dispersions apart from its influence on the energy gap (see Equation (8)). It was chosen to take *E_g_* as the fitting parameter, and to arbitrary put Δ_2_ =  0. In this way, the fitting parameters are *E_g_*, *v_z_*, *v*_∥_ and Δ_1_ (or *u*, or *v*_1_ as $v_{1}=\sqrt{1-u^{2}}\;$). They are listed in the Supporting Information. The parameter Δ_1_ was accurately deduced as it was the only parameter responsible for the well‐resolved band spin‐splitting. For Δ_2_ =  0, Equations (6) and (7) give *uv*_1_ *E_g_* = Δ_1_  so that the Rashba parameter α_*R*_ introduced above writes:

(13) $$\begin{array}{c} \;\alpha_{R}=\alpha_{1,∥}\;=\;2uv_{1}\hbar \;v_{∥}=\;4\hbar v_{∥}\frac{\Delta_{1}}{E_{g}}\; \end{array}$$

Δ_1_ has the form of an interband optical deformation potential Ξ_*o*_ and writes $\Xi_{o}\sqrt{3}\delta /4$. Thus, one retrieves Equation (3).

## Conflict of Interest

The authors declare no conflict of interest.

## Supporting information

Supporting Information

## Data Availability

The data that support the findings of this study are available from the corresponding author upon reasonable request.
