# Supplementary material for: A Novel Ferroelectric Rashba Semiconductor
Source: Adv Mater. 2023 Dec 24;36(13):2310278. doi: 10.1002/adma.202310278 (PMC11475528; doi:10.1002/adma.202310278)
Supplement: Supplementary file 1 — Supporting Information [file ADMA-36-2310278-s001.pdf]

ADVANCED MATERIALS

Supporting Information

for *Adv. Mater.*, DOI 10.1002/adma.202310278

A Novel Ferroelectric Rashba Semiconductor

Gauthier Krizman, Tetiana Zakusylo, Lakshmi Sajeev, Mahdi Hajlaoui, Takuya Takashiro, Marcin Rosmus, Natalia Olszowska, Jacek J. Kołodziej, Günther Bauer, Ondrej Caha and Gunther Springholz*

<u>Supporting Information for</u>:

A Novel Ferroelectric Rashba Semiconductor

G. Krizman, T. Zakusylo, L. Sajeev, M. Hajlaoui, T. Takashiro, M. Rosmus, N. Olszowska, J. J. Kołodziej, G. Bauer, O. Caha, G. Springholz

<u>This supporting information contains</u>:

1. Temperature dependent X-ray diffraction details.
2. The ARPES data obtained on the $Pb_{0.94}Ge_{0.06}Te$ and the $Pb_{0.975}Ge_{0.025}Te$ 9 nm-thick QWs.
3. ARPES spectra with enhanced contrast to estimate the band gap.
4. The ARPES experiments performed at the $\bar{\Gamma}$ point for each of the investigated samples and 1st derivative of the ARPES spectra at the $\bar{\Gamma}$ point to resolve the conduction states.
5. The list of the $\boldsymbol{k}.\boldsymbol{p}$ parameters used for the calculation of the QW energy spectra

1. Temperature dependent X-ray diffraction details

The complete analysis of the temperature dependent XRD data of the $Pb_{1-x}Ge_xTe$ thick film samples is summarized in Figure S1 that complements the data shown in Fig. 3 of the main manuscript. Figure S1(a) shows the measured in-plane lattice parameters of the layers as a function of temperature, evidencing a monotonous decrease with temperature that essentially follow the temperature dependence of the BaF_2 substrate caused the thermal expansion coefficient. In contrast, as shown in Fig. 3(e) of the manuscript, the out-of-plane lattice parameter shows a strong increase at and below the FE transition temperature, which induces a pronounced rhombohedral distortion along the [111] direction perpendicular to the surface. This leads naturally to a significant decrease of the lattice unit angle as is shown below in Fig. S1(b).

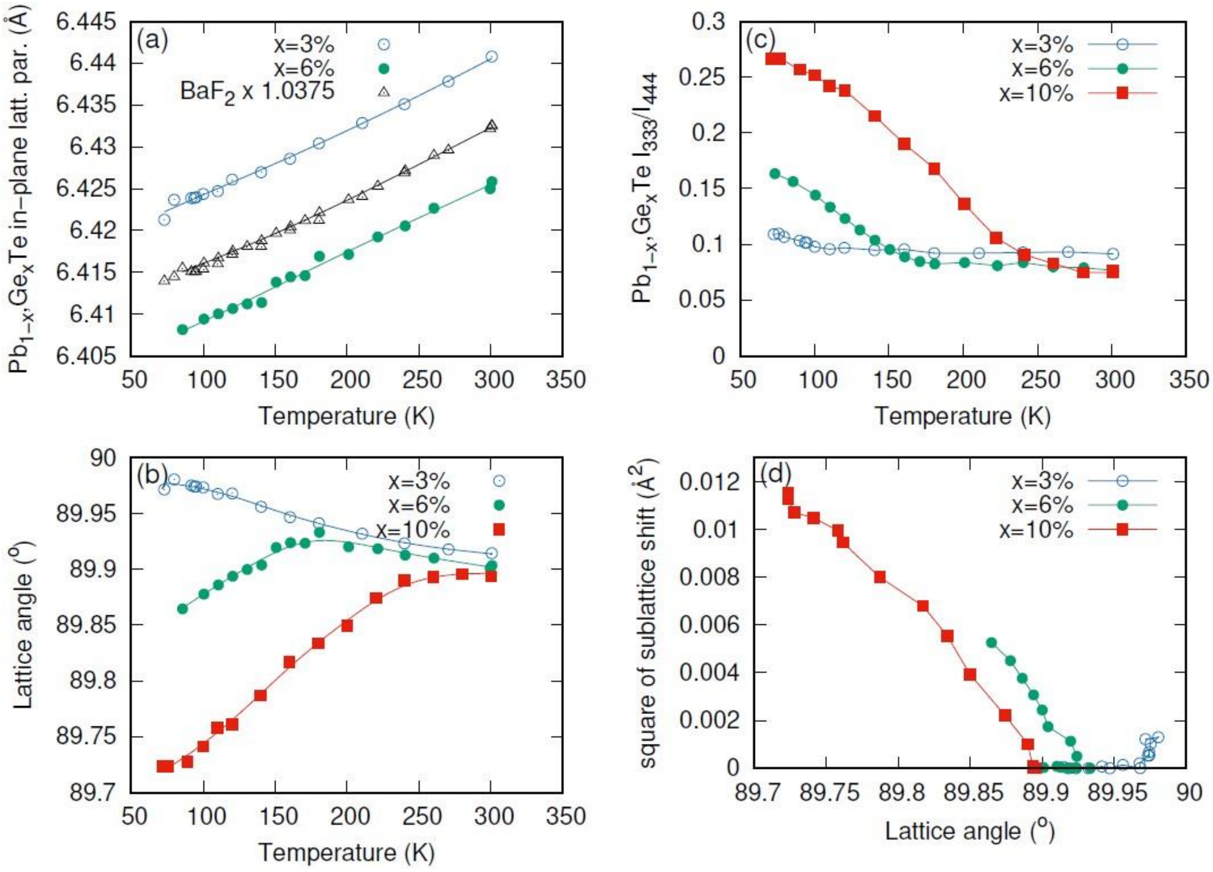

Figure S1. (a) Temperature dependence of the $Pb_{1-x}Ge_xTe$ in-plane lattice parameter deduced from asymmetric reciprocal space maps. **(b)** Rhombohedral lattice angle of $Pb_{1-x}Ge_xTe$ as a function of temperature. **(c)** Temperature evolution of the intensity ratio between the (333) and (444) Bragg reflection peaks. **(d)** Linear relation between the square of the sublattice shift δu^2 and the rhombohedral lattice angle in the FE phase.

To determine the anion/cation sublattice shift associated with the FE phase transition and the creation of the intrinsic electric dipole, we evaluate the intensity of the x-ray diffraction peaks based on standard kinematical theory of x-ray scattering, within which the intensity of the diffraction peaks is proportional to the square of the absolute value of the unit cell structure factor $I(\vec{Q}) = |F(\vec{Q})|^2$. The structure factor equals the sum over atoms in the unit cell according to:

$$F(\vec{Q}) = \sum_j f_j(Q) e^{-i\vec{Q}\cdot\vec{r}_j} e^{-B_j(T)Q^2}$$

where $\vec{Q}$ is scattering vector, f_j atomic form factor of j-th atom, $\vec{r_j}$ its position within unit cell, and $B_j(T)$ is thermal Debye-Waller factor. Due to atomic thermal vibrations the intensity of diffraction decreases with increasing temperature, which is accounted for by the Debye-Waller factor. If the relative atom positions within the unit cell are constant, the intensity ratio between different (hkl) peaks is essentially constant as a function of temperature, whereas at the FE/PE phase transition the ratio strongly changes because the anions and cations within the unit cells get shifted with respect to each other. This effect is demonstrated by Fig. S1(c), where the measured temperature dependence of the (333) and (444) Bragg peak intensities is presented for three PbGeTe layers with different compositions, demonstrating that a certain critical temperature, the ratio strongly increases and changes its slope. This is a clear shift of the shift of the atom positions within the unit cell.

In the cubic rock-salt structure above T_C, the structure factors of even order diffractions (222) and (444) are proportional to sum of the atomic form factors, since the phase term $e^{-i\vec{Q}\cdot\vec{r_j}}$ equals 1 for all atoms. For the *odd* order (*hkl*) diffractions, the phase terms have opposite signs for cations and anions positions and thus, the structure factor of the odd order diffractions is proportional to the difference of the cationic and anionic form factors. The onset of the ferroelectric lattice distortion leads to a change of the phase terms and thus, the intensity of the odd order diffraction is most sensitive to relative displacement of the cationic and anionic atoms. Substituting the displacement of anionic and cationic atoms of δ (defined in the main text) in the structure factor equation, we obtain for the intensity of odd order diffraction

$$I(Q) = (f_{Pb} - f_{Te})cos^2(2\pi(h+k+l)\delta) - (f_{Pb} + f_{Te})sin^2(2\pi(h+k+l)\delta),$$

where we have neglected the Debye-Waller factor. For the quantitative determination of the underlying displacements δ and thus δu, we have corrected the (333) diffraction intensity for a Debye-Waller factor calculated using intensities of (222) and (444) diffractions and used the equation above to then calculate the temperature dependence of the anion / cation lattice shift δu as presented in Fig. 3(f) of the manuscript. Furthermore, in Fig. S1(d) we show how the sublattice shift δ relates to the rhombohedral lattice angle, revealing that it scales quadratically with the change of the lattice angle as theoretically predicted[1,2].

2. ARPES data obtained on the $Pb_{0.94}Ge_{0.06}Te$ and the $Pb_{0.975}Ge_{0.025}Te$ 9 nm-thick QWs

Figure S2 and S3 show the temperature dependent ARPES spectra obtained for the other two PbGeTe QW samples. Figure S2 shows that data for the 9 nm-thick $Pb_{0.94}Ge_{0.06}Te$ QW at the $\overline{\mathrm{M}}$-point and at four different temperatures. A temperature-dependent Rashba splitting emerges at 160 K and increases as the temperature is decreased. Therefore, the ferroelectric phase transition is clearly evidenced with a critical temperature between 160 K and 200 K.

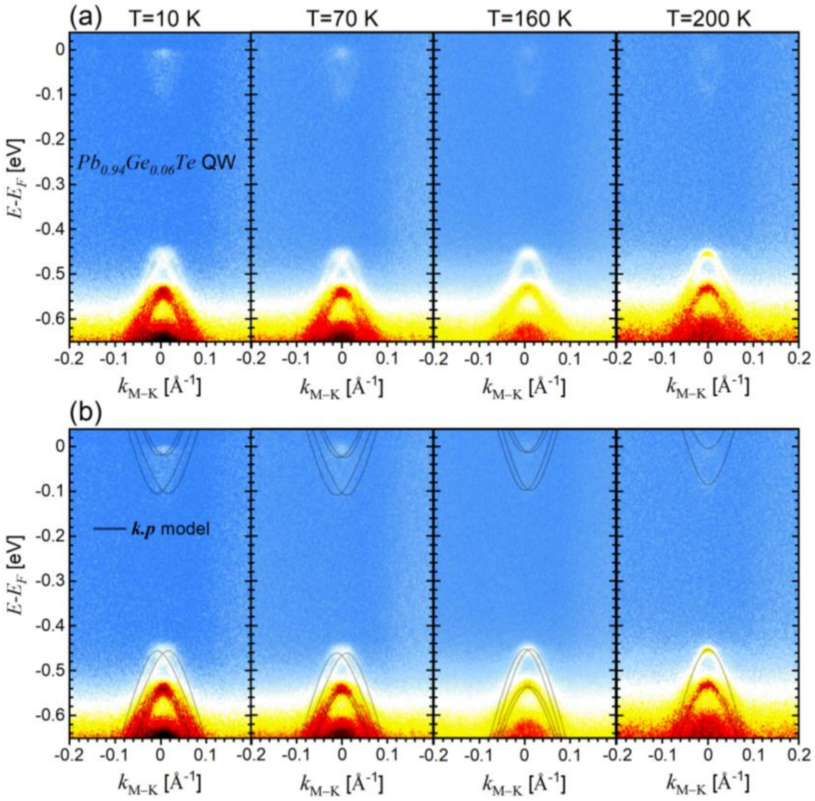

Figure S2. (a) ARPES spectra measured for the $Pb_{0.94}Ge_{0.06}Te$ 9 nm-thick QW at the $\overline{\mathrm{M}}$-point at different temperatures. **(b)** Same spectra with the superimposed fits of the quantized subbands calculated using the $\boldsymbol{k.p}$ model described in the Methods Section.

This data set has been fitted by the $\boldsymbol{k.p}$ model described in the main text, as shown by the solid lines in the bottom part of Fig. S2. The fits are excellent for each of the temperatures and for each of the observed confined states, allowing for an accurate determination of the band structure parameters and in particular, the temperature dependence of the Rashba parameter α_R. These parameters (band gap and Rashba parameter) are plotted in green in Figs. 5 and 6 of the main text and are listed in Section 5 of this supporting information. Similar measurements and fits have been performed for the $Pb_{0.975}Ge_{0.025}Te$ 9.5 nm-thick QW heterostructure, as shown in Fig. S3.

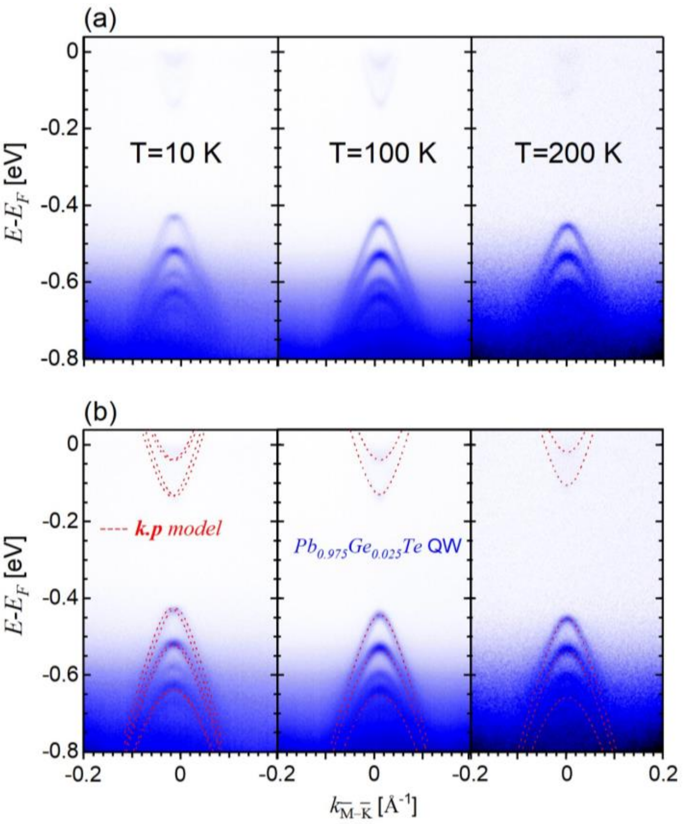

Figure S3. (a) ARPES spectra measured for the $Pb_{0.975}Ge_{0.025}Te$ 9.5 nm-thick QW with lower Ge concentration ($T_C \approx 80$ K, see Fig. 5(c) of the main text) at the $\overline{\mathrm{M}}$-point at different temperatures. **(b)** Same spectra with the superimposed fits in red dashed lines of the quantum confined subbands calculated using the $\boldsymbol{k.p}$ model.

3. ARPES spectra with enhanced contrast to estimate the band gap

Figure S4 shows the same ARPES spectra as in Fig. 5(b) of the main text, namely, the spectra around the $\overline{\mathrm{M}}$-point of the $Pb_{0.93}Ge_{0.07}Te$ QW, with strongly increased color contrast to make the conduction state more clear. This image representation allows us to determine the dependence of the band gap as a function of temperature shown in Fig. 5(c) of the main text.

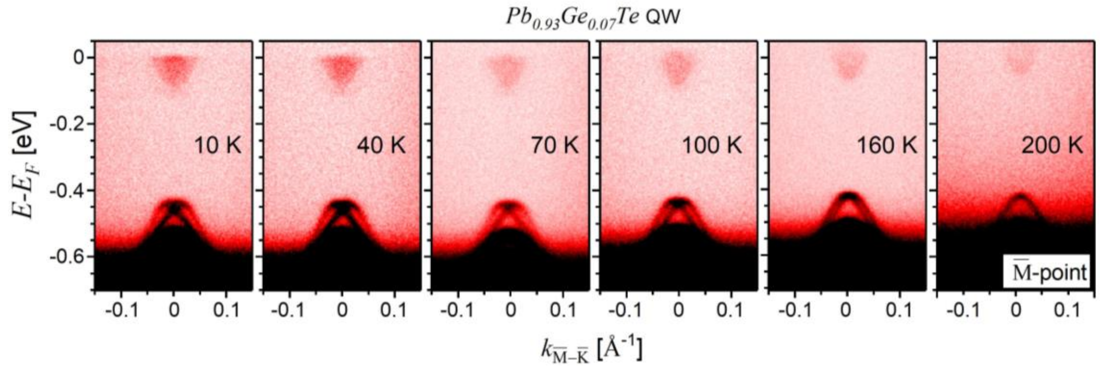

Figure S4. ARPES spectra at different temperatures taken along the $\overline{\mathrm{KMK}}$ direction of the $Pb_{0.93}Ge_{0.07}Te$ QW. The contrast has been enhanced by a factor 5 to 10 to improve the visibility and resolution of the conduction states.

4. ARPES analysis for the $\bar{\Gamma}$ point

Figure S5 shows the temperature dependence of the ARPES spectra of the ferroelectric $Pb_{0.93}Ge_{0.07}Te$ QW around the $\bar{\Gamma}$-point of the 2D (111) Brillouin zone. Similar to the data at the $\bar{M}$-point presented in Fig. 5 of the main manuscript, at low temperatures we observe a clear Rashba splitting of the quantum confined subband both in the conduction and valence band, and this splitting gradually decreases with increasing temperature and disappears above T_C of about 160 K, exactly the same as we have shown for the quantized subbands at the $\bar{M}$-point in Fig. 5(b). This is more clearly visible in the 1st derivative ARPES maps shown in the middle panel of Fig. S5.

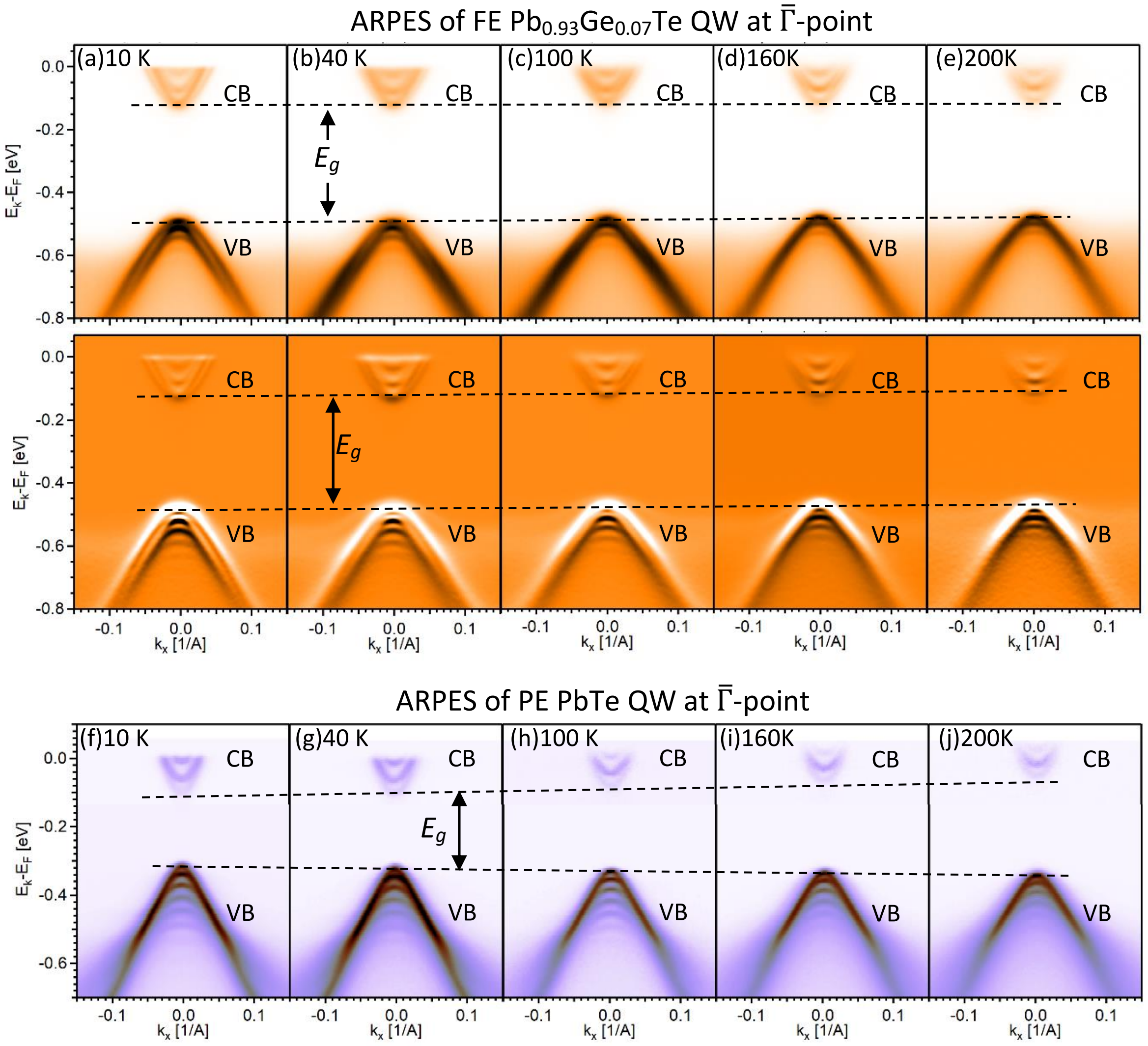

Figure S5. Temperature dependent ARPES measured at the $\bar{\Gamma}$-point for the FE $Pb_{0.93}Ge_{0.07}Te$ (**a-e**) and PE PbTe QW (**f-j**) at different temperatures from 10 to 200K. k_x denotes the momentum along the $\overline{K\Gamma K}$ direction. (a-d) Top row: ARPES maps with enhanced contrast for the conduction band; bottom row: 1st derivative along the vertical scale to enhance the visibility of the subband dispersions.

Similar data was also measured for the other QWs with $0 < x_{Ge} < 0.07$ investigated by ARPES, whose results are summarized in Fig. S6. In all cases, quantized electronic subbands are observed both at low and high temperatures, but because the energy level spacing is much narrower at the $\bar{\Gamma}$-point than at the $\bar{M}$-point due to the heavier effective confinement mass in the direction [111] at the

$\overline{\Gamma}$-point compared to the $\overline{\mathrm{M}}$-point (see Section 5 of this supporting information) the individual subbands and their Rashba splitting is less resolved. From the fit of the data, the Rashba parameter α_R at the $\overline{\Gamma}$-point is estimated in the range of 0.5 to 0.8 eV.Å for the $Pb_{0.93}Ge_{0.07}Te$ QW sample at T=10 K. Because the QW confined states are closer to each other for the $\overline{\Gamma}$ valley, the experiments lack of resolution to more precisely determine the Rashba splitting.

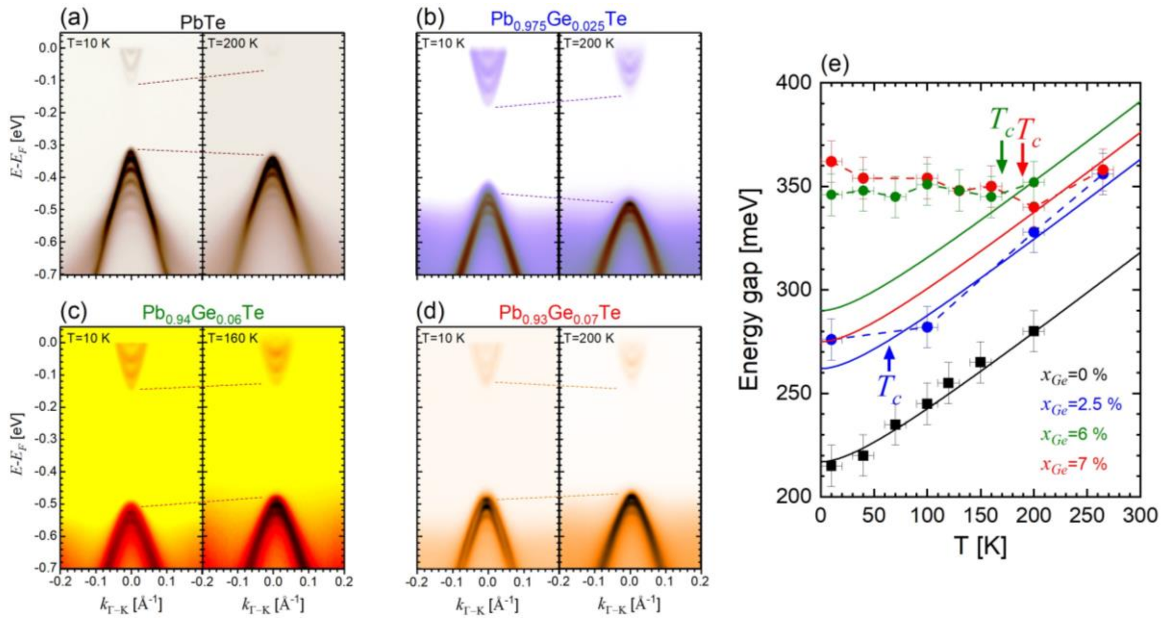

Figure S6. (a-d) ARPES spectra (left hand side) recorded at low (10 K) and high temperature (>160K) for the different $Pb_{1-x}Ge_xTe$ QW samples around the $\overline{\Gamma}$-point of the 2D Brillouin zone. The dashed lines are guide-for-the-eye to show the temperature evolution of the energy gap. **(e)** Resulting temperature dependence of the energy gap derived from the ARPES data. The solid lines denote the expected temperature dependence of the gap if the layers would remain cubic.

From the data shown in Figs. S5 and S6, the effective QW energy gaps at $k_{\parallel} = 0$ were extracted for the $\overline{\Gamma}$-point and their temperature dependence is shown in Fig. S6. They follow a similar behavior than at the $\overline{\mathrm{M}}$-point, with a clear slope change around the critical temperature of the phase transition. This is also illustrated by the dashed lines in Fig. S6, showing a reversed temperature dependence of the energy gap for the high Ge content samples.

5. The list of the $k.p$ parameters used for the calculation of the QW energy spectra

Table SI. Parameters used to fit the ARPES data obtained at the $\overline{\mathrm{M}}$-point. The thickness is fixed at 9 nm except for sample $Pb_{0.975}Ge_{0.025}Te$ that is taken to be 9.5 nm-thick. Only five parameters are used to fit the ARPES data. $E_g^{\overline{\mathrm{M}}}$ denotes the gap of the bulk band alignment.

Samples	T [K]	$E_g^{\overline{\mathrm{M}}}$ [meV]	u	$v_{\parallel}$ [x10^5 m/s]	v_z [x10^5 m/s]
PbTe	10	190	1	7.4	2.3
	70	210	1	7.4	2.3
	100	230	1	7.4	2.3
	150	245	1	7.4	2.3
	200	270	1	7.4	2.3
$Pb_{0.975}Ge_{0.025}Te$	10	215	0.995	7.1	2.75
	100	235	1	7.1	2.75
	200	270	1	7.3	2.5
	265	300	1	7.3	2.5
$Pb_{0.94}Ge_{0.07}Te$	10	310	0.975	6.5	2.1
	40	300	0.976	6.5	2.1
	70	285	0.978	6.5	2.2
	100	290	0.987	6.5	2.2
	130	300	0.99	6.5	2.2
	160	300	0.997	6.5	2.2
	200	310	1	6.5	2.2
	265	330	1	6.5	2.2
$Pb_{0.94}Ge_{0.06}Te$	10	320	0.98	6.7	2.2
	70	310	0.985	6.7	2.2
	160	290	0.995	6.5	2.2
	200	300	1	6.5	2.2

Note that the fitting parameters of PbTe are in perfect agreement with the literature[3–5]. The in-plane and out-of-plane effective masses being 0.0305 and 0.316, giving an anisotropy factor of 10.4. One can notice the decreasing anisotropy with the addition of Ge. For $Pb_{0.93}Ge_{0.07}Te$, the anisotropy factor is reduced to 8.7. Note also that $E_g^{\overline{\mathrm{M}}}$ denotes the gap of the QW *material* at the $\overline{\mathrm{M}}$-point, which correspond to the gap of the corresponding bulk $Pb_{1-x}Ge_xTe$. Figure 5(c) in the main text shows the QW gap in which the confinement energy is added.

The in-plane and out-of-plane electronic velocities ($v_{\parallel}$ and v_z respectively) are defined with respect to the [111] direction. For the oblique valleys, along the $\overline{\mathrm{KMK}}$ direction, the velocity entering into the energy-momentum dispersion is also $v_{\parallel}$[6], thus, the values found in Table SI are used to calculate the Rashba constant using $\alpha = 2uv_1\hbar v_{\parallel}$.

References:

[1] R. Clarke, *Phys. Rev. B* **1978**, *18*, 4920.

[2] G. Bauer, W. Jantsch, E. Bangert, in (Ed.: P. Grosse), Springer Berlin Heidelberg, Berlin, Heidelberg, **1983**, pp. 27–48.

[3] G. Bauer, H. Pascher, W. Zawadzki, *Semicond. Sci. Technol.* **1992**, *7*, 703.

[4] J Singleton, E Kress-Rogers, A V Lewis, R J Nicholas, E J Fantner, G Bauer, A Otero, *J. Phys. C Solid State Phys.* **1986**, *19*, 77.

[5] G. Bauer, *Narrow Gap Semicond. Phys. Appl. Proceeding Int. Summer Sch.* **1980**, *133*, 427.

[6] G. Krizman, B. A. Assaf, M. Orlita, G. Bauer, G. Springholz, R. Ferreira, L. A. de Vaulchier, Y. Guldner, *Phys. Rev. Res.* **2022**, *4*, 13179.
